# Skin Symptoms That Appeared after Fixation with a Titanium Plate in a Jaw Deformity Patient Suffering from Palmoplantar Pustulosis: A Case Report

**DOI:** 10.3390/dj11110257

**Published:** 2023-11-01

**Authors:** Fumitaka Obayashi, Koichi Koizumi, Nanako Ito, Nami Obayashi, Tomoaki Shintani, Mikihito Kajiya, Souichi Yanamoto

**Affiliations:** 1 Department of Oral Oncology, Graduate School of Biomedical and Health Sciences, Hiroshima University, Hiroshima 734-8553, Japan; fumi2390@hiroshima-u.ac.jp (F.O.); nanainc7@hiroshima-u.ac.jp (N.I.); syana@hiroshima-u.ac.jp (S.Y.); 2 Center of Oral Clinical Examination, Hiroshima University Hospital, Hiroshima 734-8551, Japan; nami-obys@hiroshima-u.ac.jp (N.O.); tshintan@hiroshima-u.ac.jp (T.S.); mkajiya@hiroshima-u.ac.jp (M.K.)

**Keywords:** palmoplantar pustulosis, jaw deformity, metal allergy, focal infections, smoking habit, oral and maxillofacial surgery, absorbable plate

## Abstract

Palmoplantar pustulosis (PPP) is a stubborn skin disease involving repeated aseptic small pustules on the palms and soles of the feet, which is triggered and exacerbated by metals and dental focal infections. There are few reports of an exacerbation of PPP symptoms after orthognathic surgery. The patient is a 40-year-old female who consulted an orthodontist at our hospital, complaining of a protruding maxilla and malocclusion. Under the diagnosis of skeletal prognathism, she underwent surgery for jaw deformity. Although no allergic symptoms were observed during the orthodontic treatment prior to surgery, postoperative scaling on the palms and soles of her feet worsened, and itching was observed on the skin, especially on the titanium plate used to secure the bone fragments. Under the diagnosis of metal allergy, treatment with steroids and vitamin D ointment failed to improve the condition, so surgery was performed to replace the metal plate with a non-metallic absorbable plate in the third postoperative month. Afterwards, the pruritus resolved, and erythema and scale on the palms and soles nearly disappeared. In the present case, though, oral bacterial infection, a past history of smoking, and stress from surgery were also considered to be possible causes of PPP exacerbation, and we concluded that one of the causes of PPP exacerbation was metal allergy from the plates or screws used to fix the bone fragments.

## 1. Introduction

Palmoplantar pustulosis (PPP) is a chronic inflammatory disease characterized by erythematous keratotic lesions with cracking, bleeding, and pain. Clinical manifestations include small, sterile pustules on the palms and soles [1,2]. The prevalence of PPP ranges from 0.050 to 0.12%, and PPP is more common in women, with the highest prevalence in those aged 50–69 years [3]. Despite the presence of specific phenotypes common to both diseases in PPP and psoriasis, it is believed that PPP should be considered a distinct disease rather than a clinical variant of psoriasis. Recently, according to the European Rare and Severet Expert Network, PPP has been considered a variant of pustular psoriasis with or without psoriasis vulgaris [3]. Synovitis, acne, pustulosis, osteitis syndrome, and pustular osteoarthritis are considered to be PPP-related diseases. Furthermore, PPP has been reported to be associated with diseases such as psychiatric disorders, thyroid-related diseases, changes in calcium homeostasis, diabetes, obesity, and dyslipidemia [3]. It has been reported that PPP is triggered and exacerbated by factors such as tonsil and odontogenic infections, smoking, stress, drug intake, and metal allergies [3,4,5,6]. Over the past 15 years, many researchers have recognized PPP as a reactive process (a subtype of systemic contact dermatitis), as there are numerous reports in the literature linking PPP to metal allergies (nickel, iron, cobalt, zinc, and copper) [6]. However, the pathology and etiology of PPP, as well as its relationship with other diseases, remain unclear.

In a retrospective study of PPP patients, more than 60% of patients showed symptom relief after dental treatment, suggesting that oral infections affect the clinical outcome of PPP [7]. A wide variety of microorganisms inhabit the oral cavity, and abnormalities of the oral microflora have been reported in patients with inflammatory diseases such as inflammatory bowel disease, rheumatoid arthritis, celiac disease, atopic dermatitis, and immunoglobulin A nephropathy, with different oral bacterial flora reported before and after dental treatment in patients with PPP.

In addition to malocclusion and jawbone morphologic abnormalities, patients with jaw deformities often exhibit jaw function that requires surgical orthodontic treatment [8]. The surgery is generally performed after the age of 17 to 18, when the growth and development of the jawbone is completed, and is considered to be appropriate until the age of 25. Therefore, there are few opportunities to perform surgery on jaw deformity patients suffering from PPP. Orthognathic surgery for PPP patients could theoretically exacerbate symptoms due to oral bacterial infection of the surface of the bone fragments or changes in the bacterial flora, metallic orthodontic materials, metal plates, or screws used to secure the bone fragments, as well as stress on intra- and postoperative pain and intermaxillary fixation, but few reports exist on the details of this problem [9].

In this case, an allergic-like reaction was observed postoperatively despite the use of a titanium plate, which is considered less likely to cause allergy, and a preoperative patch test for metal allergy was performed and confirmed negative for all antigens [10]. This is a case report of PPP exacerbation after surgery for jaw deformity, with symptoms improving after the metal plates were removed. In addition, the discussion was carefully considered with regard to the cause of the PPP exacerbation.

## 2. Case Report

**Figure 1:**
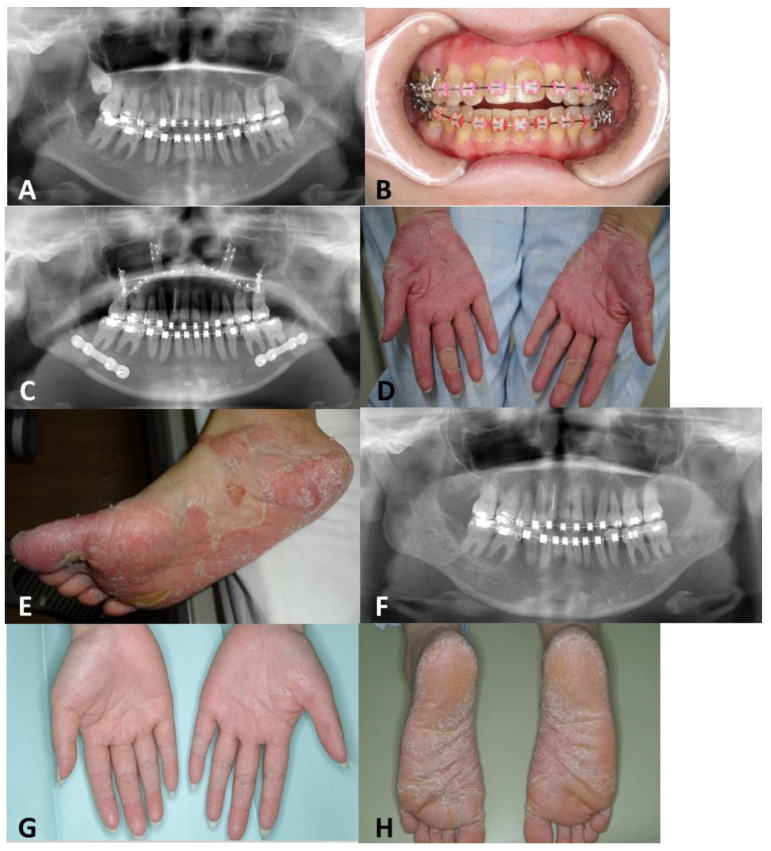
Preoperative and postoperative panoramic X-ray and photographs of palms and soles. (**A**,**B**) Preoperative panoramic X-ray and intraoral photographs are shown. (**C**) Osteotomies of the maxilla and mandible were performed and fixed with titanium plates and screws. (**D**,**E**) Symptoms of PPP worsened on the palms and soles. (**F**) Titanium plates were removed and re-fixed with absorbable plates. (**G**,**H**) After plate replacement surgery, the dermatologic symptoms on her palms and soles improved.

A 40-year-old woman visited the Department of Orthodontics and Oral and Maxillofacial Surgery at Hiroshima University Hospital for maxillary prognathism and malocclusion, where she was diagnosed with skeletal maxillary prognathism and surgical orthodontic treatment was planned. She had PPP, a metal allergy to copper (Cu), Basedow’s disease, and a history of smoking. One year ago, she was diagnosed with PPP (Psoriasis Palmoplantar Pustulosis Area and Severity Index was 1) by a dermatologist and was treated with topical steroids and active vitamin D3. Since then, there had been no exacerbation of PPP symptoms. Preoperative skin patch tests containing Cu for dental metals were negative (Supplementary Table S1). A patch test was performed with the metal reagent (Torii Pharmaceutical Co., Ltd., Tokyo, Japan) using Finn Chamber (Epitest Ltd., Oy, Tuusula, Finland) and Scanpor Tape (NORGESPLASTER A/S, Oslo, Norway). A chamber was patched on the patient’s back for 48 h. Results were observed after 48 h, 72 h, and 1 week based on the criteria of the International Society for Contact Dermatitis. Fulfilling the International Contact Dermatitis Research Group criteria or higher at 72 h was regarded as positive. Preoperative orthodontic treatment was completed without the appearance of allergy symptoms. Preoperative panoramic X-rays and intraoral photographs are shown (Figure 1A,B). The preoperative vital signs were blood pressure 140/78 mmHg, body temperature 37.4 °C, and heart rate 89 beats/min. Before and after surgery, scaling and tooth brushing instruction was performed by a dental hygienist as perioperative oral care.

Le Fort I osteotomy and a Wassmund maxillary alveolar osteotomy were performed on the maxilla, and a sagittal split ramus osteotomy was carried out on the mandible under general anesthesia [8]. Titanium miniplates were used for maxillary bone fixation, whereas 2.0 mm locking plates and corresponding screws were used for mandibular bone fixation (Figure 1C). The operative time was 5 h and 27 min, and the blood loss was 360 mL. Pruritus developed in the skin around the titanium plates used to fix the bone fragments on the 14 days after surgery. Although no obvious local signs of infection were observed, her body temperature was 38.9 °C and C-reactive protein (CRP) was 8.3 mg/dL the day after surgery. Erythema, pustules, and epidermal desquamation on the palms and soles were observed one week postoperatively, and a dermatologist at our hospital diagnosed PPP exacerbation (Figure 1D,E). Symptoms persisted until three months postoperatively despite treatment with topical steroids, active vitamin D3, biotin, and butyric acid bacteria to improve the intestinal microbiota. Vital signs before the second surgery were blood pressure 135/96 mmHg, body temperature 37.1 °C, and heart rate 72 beats/min under thiamazole administered by the internist. Under a diagnosis of metal allergy due to titanium miniplates and screws used to secure bone fragments in the upper and lower jaw, the plates and screws were removed and refixed with resorbable (or bioabsorbable) plates and screws (poly-L lactic acid/Hydroxy apatite) (Super-Fixsorb, Takiron Co., Osaka, Japan) (Figure 1F). The second operative time was 4 h and 46 min, and the blood loss was 336 mL. Postoperatively, no problems were seen compared to after the first surgery.

The composition of the metal plates and screws that were removed was examined because some materials had unknown metal content. As a method, the surfaces of the removed metal plates and screws were abraded with a silicon point, and then the metal elements contained were analyzed using an X-ray fluorescence metal element analyzer (Horiba, Ltd., Kyoto, Japan). The analysis results are shown in Table 1. Orthodontic appliances placed on teeth from preoperative orthodontic treatment contained chromium, iron, nickel, and molybdenum. In addition to titanium, the removed metal plates and screws contained metallic elements of aluminum, vanadium, and iron. After plate replacement surgery, the pruritus disappeared and dermatologic symptoms in the palms and soles improved (Figure 1G,H). Three years postoperatively, there was no recurrence of symptoms. Pre and postoperative laboratory data are shown in Table 2. The counts of red blood cells were mildly elevated but not clinically problematic, and no other laboratory findings were abnormal.

## 3. Discussion

Although there are various factors related to PPP exacerbations, their relevance to this case is discussed below.

First, the patient had a smoking history, which may have been the cause of the PPP exacerbation. High rates of current or past smoking (42–100%) have been reported in PPP patients [11,12]. Nicotine has been suggested to act on sweat glands and keratinocytes, increasing keratinization and promoting neutrophilic inflammation [13]. Recently, Kobayashi and colleagues demonstrated a direct relationship between PPP severity and smoking in female PPP patients. A direct relationship was demonstrated [14]. Although this patient has a history of smoking, she was not smoking at the time of our visit, and it is impossible to gauge the extent to which past smoking has affected the exacerbation of PPP.

Second, it has been reported that PPP patients experience an exacerbation of their lesions during periods of stress [3]. Usually, after surgery under general anesthesia, many patients complain of a sore throat caused by the intubation tube. In addition, after surgery for jaw deformity, the upper and lower jaws are immobilized in an occluded position to rest the fixed bone fragments, making it impossible for the patient to open the mouth. Patients are also under a lot of stress because they have to eat through a tube inserted through the nose to the stomach for nutritional intake. In this case, the patient was also under stress.

Third, regarding metal allergy, about 69.8% of Japanese PPP patients tested positive for the patch test [15]. Titanium has high biostability and is thought to be hypoallergenic [10]. Brunasso et al. reported that 122 of 519 PPP patients had both PPP and contact allergy; no allergen was reported in 36 cases; allergens were reported in 86 cases of PPP and contact allergy; and 67 cases had a metal allergy [6]. On the other hand, contact allergy was associated with other non-metal allergens in 19 cases. Four cases of PPP who developed metal allergy with high blood mercury levels improved with diet and/or chelation therapy, suggesting a close relationship between metal exposure and PPP. These results indicate that metal allergy is one of the causes of the exacerbation of PPP in this case. Her history interview revealed a history of metal allergy to Cu, but a preoperative patch test was negative. We examined the metallic elements in the orthodontic device and in the removed plates and screws with an analyzer. Elemental analysis also showed that the plates and screws used for fixation in the initial surgery did not contain Cu. Discrepancies between patch test results and biological responses to metals are often observed, and the metal screws for fixing contain trace amounts of aluminum, vanadium, and iron, which may be allergens. It is necessary to consider the reason why the PPP appeared after the osteotomy of the jawbone, even though no exacerbation of PPP was observed with wires and hooks during the preoperative correction. In response to this contradiction, we think that the tip of the screw for fixing the bone fragment was located in the bone marrow, with abundant blood flow, thus facilitating allergen sensitization.

Fourth, we discuss the relationship between PPP and focal infections. PPP has been attributed not only to tonsil infections but also to periapical lesions, periodontitis, pericoronitis, and sinusitis [4,7]. More than 60% of Japanese PPP patients report symptom improvement after treating a dental infection (periodontitis or endodontic treatment) [15]. Tanimoto et al. reported that an enhanced keratin-specific immune response in infectious foci may contribute to its pathogenesis [16]. PPP patients reportedly have different oral flora from healthy controls [17]. Akiyama et al. analyzed the oral flora before and after dental treatment and among healthy controls [7]. They found that *Proteobacteria* were the most abundant in the healthy control group, while *Selenomonas* was predominant in the post-dental treatment group. As a genus specific to PPP, *Eggethellacea*, which is frequently isolated from bacteremia cases, was detected in PPP patients. The oral microflora was not analyzed in this case, and will be the subject of future studies. In this case, bacteremia from the surface of the bone fragments, and upper respiratory tract infection or pharyngitis due to endotracheal intubation could have aggravated the PPP. CRP was 8.3 mg/dL the day after surgery. It often elevates to this level due to osteotomies of the maxilla and mandible, which are highly invasive surgeries, and we do not believe that elevated CRP is a direct trigger for the exacerbation of PPP symptoms. However, there are many bacteria in the oral cavity, and given the size of the surgical wound and the duration of surgery, it cannot be denied that oral bacteria have some influence on the exacerbation of PPP.

Finally, paradoxical adverse events (PAEs) during anti-TNF-α therapy for psoriasis have been reported in 2–5% of patients, and PPP is the most common skin manifestation of PAEs [18]. However, it is not considered an exacerbating factor because no anti-TNF-α based pharmacotherapy was used in this case.

Smoking, stress, metal allergy, and infection were considered to be responsible for the exacerbation of PPP in this case. It is difficult to identify risk factors for PPP exacerbations; of course, it is not incorrect to assume that the synergistic effect of these four causes has exacerbated PPP. Not only the physical contact of wires and other metals with mucous membranes, but also the attachment of orthodontic appliances, may make plaque control more difficult, resulting in the growth of periodontal disease and caries-causing bacteria, etc., which may cause an exacerbation of PPP. No exacerbation of PPP was observed with the contact of wires or metal brackets with the oral mucosa in preoperative orthodontic treatment. However, an exacerbation of PPP was observed when osteotomies were performed. Since the symptoms of PPP were alleviated by replacement with absorbable plates, we concluded that one of the causes of PPP exacerbation was metal allergy from the plates or screws used to fix the bone fragments. This report is also useful for preventing the exacerbation of PPP when performing surgery for jaw deformities in PPP patients.

## 4. Conclusions

In surgical cases of jaw deformities with PPP, the following are necessary to understand the pathogenesis of PPP and to eliminate the causes of PPP exacerbation. Perioperative oral care and tooth brushing instruction, including during preoperative orthodontic treatment, are necessary not only to control oral bacteria, but also to improve lifestyle habits such as smoking cessation. In addition, the use of absorbable non-metallic plates to fix bone fragments should be considered in cases of pre-existing or suspected metal allergy.

## Figures and Tables

**Table 1:** Elemental analysis of the titanium miniplates and screws for surgery and the wire/hook for the orthodontic treatment.

Miniplate/Screw	Ti	Al	V	Fe				
L-Plate	+	-	-	+				
-Screw	+	-	+	+				
Le Fort Plate	+	-	-	+				
-Screw	+	+	+	+				
Locking Plate	+	+	-	+				
-Screw	+	+	+	-				
**Wire/Hook**	**Co**	**Cr**	**Ni**	**Fe**	**Mo**	**Mn**	**Cu**	**Si**
Chromium Cobalt Wire	+	+	+	+	+	+	-	+
Stainless Steel Wire	-	+	+	+	+	-	+	+
Ligature Wire	-	+	+	+	+	-	-	-
Surgical hook	-	+	+	+	-	-	-	+

There was no description of contained metals in the manual. Ti, titanium; Al, aluminum; V, vanadium; Fe, iron; Co, cobalt; Cr, chromium; Ni, nickel; Mo, molybdenum; Mn, manganese; Cu, copper; Si, silicon. “+” means that the element is included, while “-” indicates that it is not included.

**Table 2:** Results of laboratory data.

	Before the First Surgery	Before the Second Surgery	After the Second Surgery
WBC (×10^3^/µL)	9.8	7.7	7.9
RBC (×10^6^/µL)	4.9	4.7	4.6
PLT (×10^3^/µL)	239	237	260
NE (%)	71	65	68
AST (IU/L)	22	13	14
ALT (IU/L)	22	6	9
Albumin (g/dL)	3.7	3.9	4.1
Creatinine (mg/dL)	0.32	0.5	0.62
BUN (mg/dL)	8.5	6	7.1
Triglyceride (mg/dL)	N/A	200	180
HDL cholesterol (mg/dL)	N/A	47	36
LDL cholesterol (mg/dL)	N/A	127	139
CRP (mg/dL)	0.21	0.32	0.43

## Data Availability

The data presented in this study are available on request from the corresponding author. Publicly available datasets were analyzed in this study.

## Supplementary Materials

The following supporting information can be downloaded at: https://www.mdpi.com/article/10.3390/dj11110257/s1, Supplementary Table S1: Results of patch test for metal allergy.

## References

[1] Heidemeyer K., May Lee M., Cazzaniga S., Yawalkar N., Naldi L. (2023). Palmoplantar Pustulosis: A Systematic Review of Risk Factors and Therapies. Psoriasis.

[2] Zhang M., Hua L., Hong S., Sun X., Zhou Y., Luo Y., Liu L., Wang J., Wang C., Lin N. (2023). Efficacy and safety of biological agents to treat patients with palmoplantar pustulosis: A systematic scoping review. Int. Immunopharmacol..

[3] Brunasso A.M.G., Massone C. (2021). Recent advances in palmoplantar pustulosis. Fac. Rev..

[4] Kikuchi N., Yamamoto T. (2013). Dental infection as a triggering factor in palmoplantar pustulosis. Acta Derm. Venereol..

[5] Takaoka Y., Akiba Y., Nagasawa M., Ito A., Masui Y., Akiba N., Eguchi K., Miyazawa H., Tabeta K., Uoshima K. (2022). The relationship between dental metal allergy, periodontitis, and palmoplantar pustulosis: An observational study. J. Prosthodont. Res..

[6] Brunasso Vernetti A.M.G., Puntoni M., Massone C. (2019). Palmoplantar Pustulosis and Allergies: A Systematic Review. Dermatol. Pract. Concept..

[7] Akiyama Y., Minabe M., Inada J., Nomura T., Takahashi S., Ishihara K., Kouno M. (2021). The oral microbial composition and diversity affect the clinical course of palmoplantar pustulosis patients after dental focal infection treatment. J. Dermatol. Sci..

[8] Koizumi K., Shintani T., Yoshimi Y., Higaki M., Kunimatsu R., Yoshioka Y., Tsuga K., Tanimoto K., Shiba H., Toratani S. (2022). Impact of Maximum Tongue Pressure in Patients with Jaw Deformities Who Underwent Orthognathic Surgery. Diagnostics.

[9] Oda A., Yoshida K., Uno T., Yoshinaka T., Mukai A., Irifune M. (2015). A Case with Deteriorating Palmoplantar Pustulosis and Hyperthyroidism after Simultaneous Bimaxillary Orthognathic Surgery. J. Jpn. Dent. Soc. Anesthesiol..

[10] Nakagawa M., Yagami A., Shimizu Y., Washimi Y., Suzuki K., Matsunaga K. (2009). Study of 10 Cases Suspected of Skin Disorders Due to Allergic Reaction to Metallic lmplant. J. Environ. Dermatol. Cutan. Allergol..

[11] Michaëlsson G., Gustafsson K., Hagforsen E. (2006). The psoriasis variant palmoplantar pustulosis can be improved after cessation of smoking. J. Am. Acad. Dermatol..

[12] Flores-Balderas X., Peña-Peña M., Rada K.M., Alvarez-Alvarez Y.Q., Guzmán-Martín C.A., Sánchez-Gloria J.L., Huang F., Ruiz-Ojeda D., Morán-Ramos S., Springall R. (2023). Beneficial Effects of Plant-Based Diets on Skin Health and Inflammatory Skin Diseases. Nutrients.

[13] Hagforsen E., Michaëlsson K., Lundgren E., Olofsson H., Petersson A., Lagumdzija A., Hedstrand H., Michaëlsson G. (2005). Women with palmoplantar pustulosis have disturbed calcium homeostasis and a high prevalence of diabetes mellitus and psychiatric disorders: A case-control study. Acta Derm. Venereol..

[14] Kobayashi K., Kamekura R., Kato J., Kamiya S., Kamiya T., Takano K., Ichimiya S., Uhara H. (2021). Cigarette Smoke Underlies the Pathogenesis of Palmoplantar Pustulosis via an IL-17A-Induced Production of IL-36γ in Tonsillar Epithelial Cells. J. Investig. Dermatol..

[15] Kouno M., Nishiyama A., Minabe M., Iguchi N., Ukichi K., Nomura T., Katakura A., Takahashi S. (2017). Retrospective analysis of the clinical response of palmoplantar pustulosis after dental infection control and dental metal removal. J. Dermatol..

[16] Tanimoto Y., Fukuyama S., Tanaka N., Ohori J., Tanimoto Y., Kurono Y. (2014). Presence of keratin-specific antibody-forming cells in palatine tonsils of patients with pustulosis palmaris et plantaris (PPP) and its correlation with prognosis after tonsillectomy. Acta Otolaryngol..

[17] Kouno M., Akiyama Y., Minabe M., Iguchi N., Nomura T., Ishihara K., Takahashi S. (2019). Dysbiosis of oral microbiota in palmoplantar pustulosis patients. J. Dermatol. Sci..

[18] Conrad C., Di Domizio J., Mylonas A., Belkhodja C., Demaria O., Navarini A.A., Lapointe A.K., French L.E., Vernez M., Gilliet M. (2018). TNF blockade induces a dysregulated type I interferon response without autoimmunity in paradoxical psoriasis. Nat. Commun..

