# Supplementary material for: Skin Symptoms That Appeared after Fixation with a Titanium Plate in a Jaw Deformity Patient Suffering from Palmoplantar Pustulosis: A Case Report
Source: Dent J (Basel). 2023 Nov 1;11(11):257. doi: 10.3390/dj11110257 (PMC10670456; doi:10.3390/dj11110257)
Supplement: Supplementary file 1 [file dentistry-11-00257-s001.zip › dentistry-2599381-supplementary.pdf]

Supplementary Table S1

Results of patch test for metal allergy

Metal allergens	Concentration	Reaction
aluminium chloride	2%	−
gold(III) chloride	0.2%	−
tin(III) chloride	1%	−
iron(III) chloride	2%	−
hexachloro platinum (IV)	0.5%	−
palladium chloride	1%	−
indium trichloride	1%	−
iridium tetrachloride	1%	−
zinc chloride	2%	−
manganese(II) chloride	2%	−
sliver bromide	2%	−
potassium dichromate	0.5%	−
cobalt(III) chloride	2%	−
copper(II) sulfate	1%	−
mercury(II) chloride	0.05%	−
nickel(II) sulfate	5%	−
chromium(III) sulfate	2%	−

A patch test was performed with the metal reagent (Torii Pharmaceutical Co., LTD., Tokyo, Japan) using Finn Chamber (Epitest Ltd. Oy, Tuusula Finland) and Scanpor Tape (NORGESPLASTER A/S, OSLO. Norway). A chamber was patched on the patient’s back for 48 hours. Results were observed after 48 hours, 72 hours, and 1 week based on the criteria of the International Society for Contact Dermatitis. Fulfilling the International Contact Dermatitis Research Group criteria or higher at 72 hours was regarded as positive.
